# “Under Pressure”, Hair Braids in the Critically Ill

**Published:** 2014-09-29

**Authors:** Jonathan Lam, Christopher Abraham, Thomas Resch, Stephen M. Milner, Leigh Ann Price

**Affiliations:** Johns Hopkins Burn Center, The Johns Hopkins University School of Medicine, Baltimore, Md

**Keywords:** pressure sore, hair braids, hair weave, burn, critically ill

## DESCRIPTION

**Figure 1:**
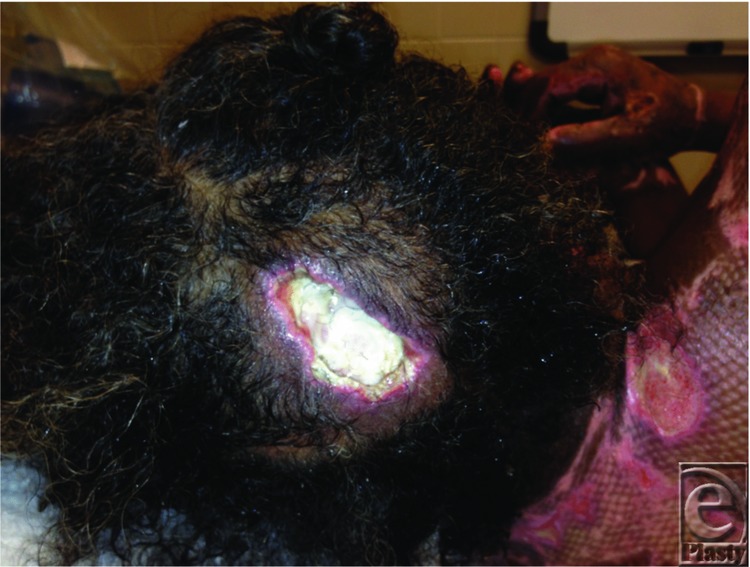
Occipital lesion.

A 56-year-old woman was admitted with 17% total body surface area deep burns. Intubated en route to the hospital for possible inhalation injury and remained intubated for 9 days due to her underlying pulmonary history. Her hospital course was complicated by an occipital lesion discovered during a routine dressing change (Fig 1).

## QUESTIONS

1. **What is the differential diagnosis of the lesion?**
2. **What is the pathophysiology behind such lesions?**
3. **What are some of the unique challenges and issues of managing these lesions in the burn unit?**
4. **What are the treatment options and prevention strategies for these lesions?**

## DISCUSSION

The differential diagnosis of the lesion can involve anything from an infectious etiology to autoimmune to iatrogenic causes. This can include, but not limited to, abscess, mycotic infection, warfarin-induced skin necrosis due to continuous Coumadin (previous history of femoral thrombus), vasculitis, pressure ulcer, pyoderma gangrenosum, and ecthyma gangrenosum. In this case, based upon the presentation and history of the lesion, the most likely diagnosis is a pressure ulcer.

Pressure ulcers are a problem that every health care institution encounters and are most often seen in patients who are immobile. These lesions commonly occur along boney prominences such as the occiput, calcaneus, scapula, elbow, ischium, greater trochanter, and sacrum. Undiagnosed and untreated pressure ulcers may result in osteomyelitis, bacteremia, cellulitis, endocarditis, and meningitis.^1^ The development of pressure ulcers involves distinct factors of both pressure and tissue tolerance. Increased pressure, whether through decreased mobility or decreased sensory perception, can cause ischemic changes in the underlying tissue bed while moisture and shear forces can affect tissue integrity.^2^ These are the important reasons for expeditiously transferring patients off hard backboards in the trauma bay. It has been documented that pressure ulcer may occur as rapidly as 2 to 6 hours.^3^ Other additional factors such as traumatic injuries, infection, nutrition, inflammation, and edema can further complicate and accelerate the progression of a developing ulcer. Particularly in burn injuries, it has been theorized that the massive release of inflammatory mediators such as prostaglandins may result in additional tissue compromise.^4^

**Figure 2:**
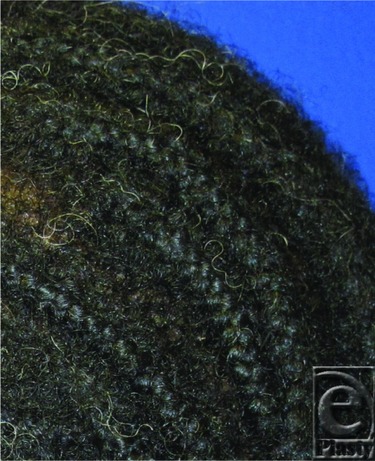
Braided hair.

The overall incidence of pressure ulcer development in critically ill patients ranges between 3.8% to 12.4%.^1^^,^^5^ Fritsch et al's study of the burn patient population reported an incidence of 4.1% among 217 patients with burn injuries.^1^ Additional factors seen in burn patients such as the loss of skin integrity, massive fluid resuscitation, edema, fascial excisions, and long-term splinting causing artificial areas of firm prominence present unique risks to the burn population.^5^ Nutrition plays a significant role in management due to the hypermetabolic nature resulting from burn injuries. Burn patients have a falsely elevated nutritional status in regard to receipt of full enteral support and even increased enteral support has been shown to falsely elevate Braden scores. In reality, major burn injuries result in significant malnutrition related to loss of serum protein hypermetabolism.^1^ In this case, the occipital ulcer most likely resulted from bands of tightly braided hair very close to the scalp that exacerbated the tension and contact pressure point and may even have caused ischemic changes (Fig 2).

Hair styled in cornrows or braids held tightly against the scalp serves as a possible nidus for infection particularly in scalp burns and is often trimmed or shaved in these instances. However, another consideration in the event of temporary hair loss is the psychological stress. Even temporary hair loss can have a significant effect on body image and psychological recovery.^6^ In a study conducted on the perception of body image in burn patients, Tagkalakis and Demiri^6^ reported that any acute change in a person's body will undoubtedly cause a temporary or permanent alteration of one's integrity regardless of sex. Females are particularly at risk, their hair has been linked to a sense of femininity, sexuality, and beauty; thus, even temporary alopecia may have a huge psychological impact.^8^

**Figure 3:**
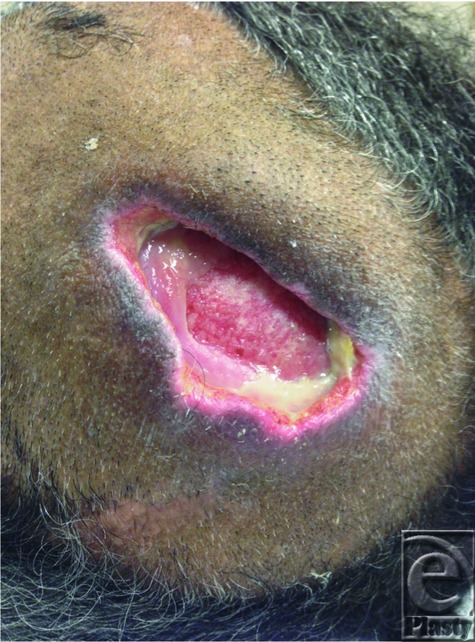
Ulceration with clean wound bed.

**Figure 4:**
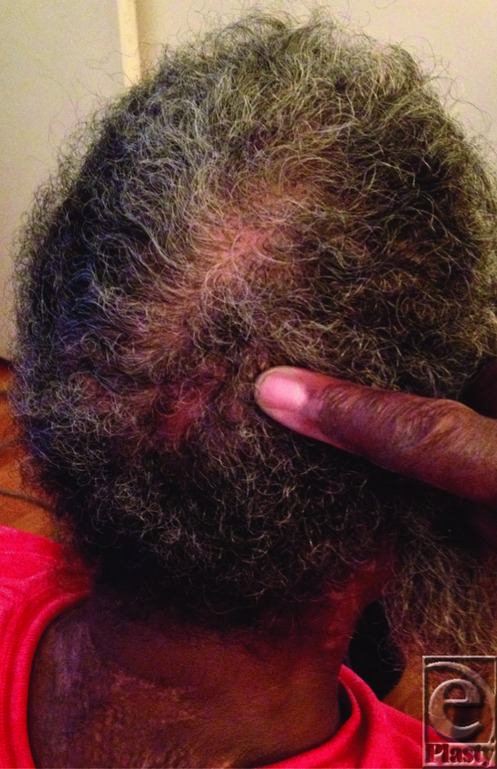
Healed wound with maturing scar and regrowth of hair.

Prevention and treatment of pressure ulcers is uniform in all populations. Initial prevention efforts should be aimed at identification of at-risk patients with utilization of specialty beds and offloading of pressure points ideally every 2 to 4 hours on the ward, as well as during any operation.^1^^,^^4^ Several methods have been developed to stage the degree of risk for the early development of pressure ulcers, most notably the Braden Scale. The Braden Scale examines 6 criteria to assess patient risk for the development of pressure ulcers, which include sensory perception, moisture, activity, mobility, nutrition, and friction/shear. Each of these criteria is rated on a scale of 1 to 4 with a score of less than 9 indicative of severe risk. In a study of patients with severe burn conducted by Lewis et al^5^ using the Braden Risk Assessment Scale, it was found that a score less than 16 was associated with an increased risk of pressure ulcer development. Having health care providers trained to properly utilize the Braden Scale produces a reliability of R = 0.99 in the early identification of at-risk patients in comparison to previously used risk assessments.^2^^,^^7^ Treatment options vary depending on the quality of the wound bed. Intact wound beds with supple granulation tissue and minimal exudate may be managed nonsurgically with topical films, hydrogels, and occlusive dressings. More contaminated wounds with moderate to significant exudate burden and minimal granulation tissue should be aggressively managed with either surgical or enzymatic debridement, followed by negative pressure wound therapy.^4^ This patient's wound had visible purulent exudate, which prompted initial management of shaving the involved area of scalp and local mechanical debridement. After attaining a clean, healthy appearing wound bed (Fig 3), negative pressure therapy was applied with complete resolution of the ulcer (Fig 4). This is an important lesson learned in a patient without a scalp burn and tightly braided hair.
